# Macrophage Clearance of Apoptotic Cells: A Critical Assessment

**DOI:** 10.3389/fimmu.2018.00127

**Published:** 2018-01-30

**Authors:** Siamon Gordon, Annette Plüddemann

**Affiliations:** ^1^College of Medicine, Graduate Institute of Biomedical Sciences, Chang Gung University, Taoyuan City, Taiwan; ^2^Sir William Dunn School of Pathology, University of Oxford, Oxford, United Kingdom; ^3^Nuffield Department of Primary Care Health Sciences, University of Oxford, Oxford, United Kingdom

**Keywords:** apoptosis, apoptotic cells, clearance of apoptotic cells, phagocytosis, macrophages

## Abstract

As the body continues to grow and age, it becomes essential to maintain a balance between living and dying cells. Macrophages and dendritic cells play a central role in discriminating among viable, apoptotic, and necrotic cells, as selective and efficient phagocytes, without inducing inappropriate inflammation or immune responses. A great deal has been learnt concerning clearance receptors for modified and non-self-ligands on potential targets, mediating their eventual uptake, disposal, and replacement. In this essay, we assess current understanding of the phagocytic recognition of apoptotic cells within their tissue environment; we conclude that efferocytosis constitutes a more complex process than simply removal of corpses, with regulatory interactions between the target and effector cells, which determine the outcome of this homeostatic process.

## Introduction

Programmed cell death, apoptosis, is closely linked to recognition and clearance by phagocytosis, resulting in anti-inflammatory and compensatory growth responses during fetal and adult life. Prolonged or incomplete containment and destruction during the apoptotic process can merge into secondary necrosis to bring about pro-inflammatory and pathological responses by phagocytes that are still poorly understood. Many aspects of this process are covered in excellent reviews by leading investigators, including Henson (1), Arandjelovic and Ravichandran (2), Nagata and Tanaka (3), Lim et al. (4), and their colleagues, which should be consulted for detailed exposition. We have previously reviewed the process of phagocytosis in relation to infection and microbial recognition (5), emphasizing its cell biology in model systems. We focus here on clearance of apoptotic cells *in situ* by heterogeneous tissue mononuclear phagocytes, in health and disease.

## A Brief History

Although current nomenclature settled around the early reports by Wyllie et al. (6), the observations of cell death in its many manifestations date back to the nineteenth century, from descriptions by Virchow, Metchnikoff, and many pathologists. Wallach et al. have provided an instructive time line of concepts of tissue injury and cell death in inflammation (7). An historic perspective of macrophages, phagocytic mechanisms, and lysosomal digestion is provided elsewhere (5, 8). The pre-eminent role of tissue macrophages in clearance was emphasized in the twentieth century, as a primary function of the reticuloendothelial system, subsequently renamed the mononuclear phagocyte system (MPS) (9). Studies in *Caenorhabditis elegans* (10) stimulated genetic dissection of apoptosis and clearance by epithelial cells in organisms that lack “professional” phagocytes; important discoveries of macrophage clearance followed in *Drosophila* and other model organisms, such as zebra fish and mice. Uptake of dead cells by non-professional phagocytes in vertebrates became overshadowed by emphasis on macrophages and related dendritic cells (DCs), although recent studies (2) have to some extent redressed the balance; turnover of photoreceptors by retinal pigment epithelia and of aberrant sperm in the testis by Sertoli cells are highly active functions of non-hematopoietic phagocytic cells, and uptake of cell corpses has also been demonstrated in epithelia, fibroblasts, astrocytes, and cancer cells, the so-called non-professional phagocytes (11). Different terms have emerged for a range of distinct though related processes, in addition to efferocytosis (12); these include necroptosis (13), pyroptosis (14), phagoptosis (15), ferroptosis (16), trogocytosis (17), and entosis (18), depending on one or other characteristic feature. Mevorach and colleagues have introduced clarity into the terminology of this expanding topic, which will be defined as relevant, below (19). Henson and Bratton (20) provided early evidence that clearance of programmed apoptotic cell death by macrophages gave rise to anti-inflammatory effects, unlike the pro-inflammatory consequences of the uptake of necrotic cells, which could follow at a further stage of programmed cell death, during infection or as a result of accidental injury. Another time line of particular interest in this area is given by Nagata and Tanaka, who pioneered the role of phosphatidyl serine (PS) and membrane lipid reorganization in the recognition of apoptotic cells (3).

The physiological role of apoptotic cell clearance by macrophages has been documented in organ development, tissue remodeling, e.g., in the uterus and mammary gland, repair and potential cell replacement following injury and, in a few species, regeneration of complex organs. In pathology, monocytes, macrophages, and DCs are important contributors to inflammation and its resolution, following disposal of necrotic corpses and subcellular constituents, e.g., during infection, innate and autoimmunity, atherogenesis, and malignancy. Many authors have considered cell death, its recognition, disposal, and regulation as central homeostatic functions of the MPS; aspects of this topic are reviewed by the various contributors to this Frontiers of Immunology collection, cited as available at the time of writing (21, 22). We shall not deal in this review with the mechanisms of cell death itself.

## Mononuclear Phagocytes are Highly Heterogeneous

The cells of the mammalian MPS constitute a widely dispersed population derived from common hematopoietic progenitors, which are distinct in the embryo and adult (23). Tissue-resident macrophage populations in the fetus are distributed from yolk sac and fetal liver precursors from mid gestation, and turn over locally to a variable extent throughout adult life (24). From birth, bone marrow-derived monocytes are recruited to replenish and supplement tissue macrophage populations in the steady state, and in response to inflammatory, metabolic, infectious, and malignant disease processes, as required. Circulating mononuclear cells contain precursors of macrophages, DCs, and osteoclasts, and subpopulations of monocytes that are characterized by distinct marker antigens and receptors (25). Recent single cell RNA analysis has revealed additional mononuclear cell subpopulations in blood without functional characterization (26). Trahtemberg and colleagues identified two human blood monocyte-derived DC subpopulations which differ in their expression of surface markers, phagocytic clearance, and responses to the uptake of apoptotic cells (27). The markedly heterogeneous mononuclear phagocytes in tissues display the ability to phagocytose particulates to a variable extent, depending on their differentiation, recruitment, and activation by tissue-specific and microbial stimuli. The ability of neutrophils which are highly phagocytic for opsonized microbes, and of eosinophils, basophils, and mast cells, to phagocytose apoptotic targets has been less studied. In addition, MPS cells variably display fluid phase, macropinocytic, and receptor-mediated endocytosis, which contribute to their clearance of solutes and smaller particulates. In response to local need, resident macrophages can migrate from adjacent reservoirs, as elegantly demonstrated through *in vivo* experiments by the groups of Wang and Kubes and Robbins, respectively, in liver (28) and heart (29). The prototypic and organ-specific phagocytic activity of the mixed populations of macrophages in different organs differ considerably, as shown by elegant clearance experiments *in vivo*, described in detail below (30, 31). Fusion of monocytes and macrophages gives rise to the formation of osteoclasts in bone and of multinucleated giant cells (MNGCs) in granulomatous diseases (32). We have demonstrated a striking difference in the phagocytosis of a range of particulates, including latex and complement-, but not antibody-opsonized sheep erythrocytes by IL4/13 cytokine-induced MNGC (33). Finally, activation of macrophages by innate and Th1- and Th2-dependent adaptive immune stimuli induces a spectrum of changes in cellular phenotype, including cytocidal and phagocytic capacity (34), hitherto mainly studied *in vitro*.

## Myeloid Cell Turnover

Myeloid leukocytes themselves undergo apoptosis and necrosis, as well as inducing these processes in a range of target cells. We therefore briefly consider aspects of their own turnover, clearance, and mechanisms of death that may be relevant to subsequent consequences. Recent studies by Yona and colleagues have established the circulation time and precursor–product relationships of different human monocyte subsets (35). Macrophages can become long-lived, terminally differentiated tissue-resident cells which remain biosynthetically active while ceasing to proliferate, but recently recruited monocytes and activated macrophages and DC themselves can undergo apoptosis within days of responding to inflammatory and infectious stimuli. By comparison, neutrophils are short lived and readily proceed from apoptosis to secondary necrosis when activated during acute inflammation; eosinophils may survive longer in tissues than usually appreciated. The microbicidal and cytocidal activities of activated neutrophils and monocyte/macrophages depend on generation of reactive radicals derived from oxygen through a respiratory burst; in addition, degranulation by neutrophils and eosinophils releases potent neutral proteinases such as elastase, as well as myeloperoxidase, to initiate cytotoxicity, whereas interferon gamma activated macrophages contribute to target cell killing by release of nitrogen metabolites generated by inducible nitric oxide synthase. Other mechanisms of myeloid cell cytotoxicity include chromatin extracellular trap (neutrophil extracellular trap) production by neutrophils and probably activated macrophages and release of tumour necrosis factor (TNF)alpha. Inflammasome activation and IL-1 release depend on selected proteolysis by cytosolic caspases. Depletion of essential amino acids such as tryptophan and arginine in their local environment contribute to cell death.

## Interactions Between Macrophages and Apoptotic Cells

**Figure 1:**
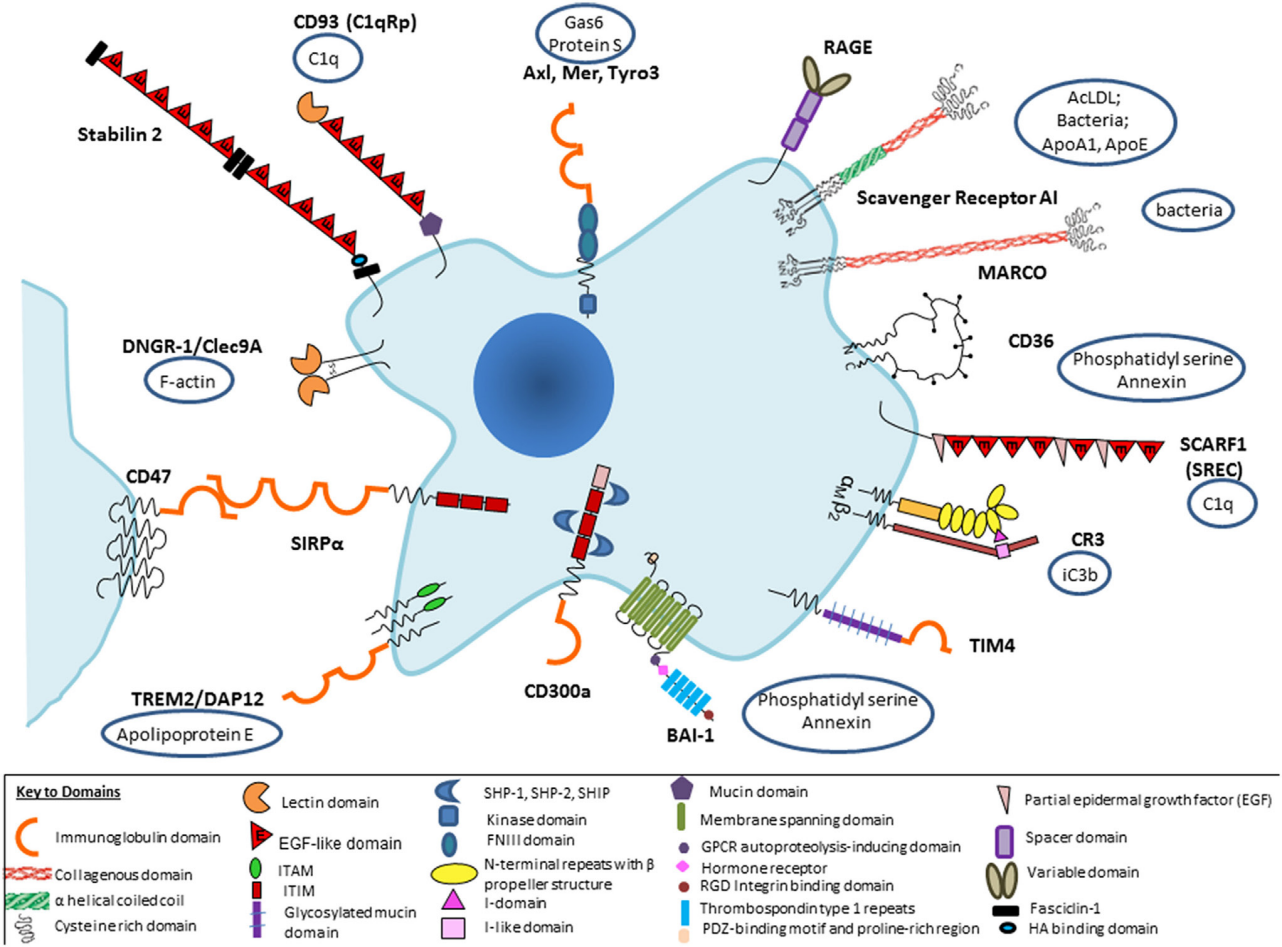
Selected macrophage phagocytic receptors and ligands implicated in apoptotic cell clearance. Receptor–ligand interactions can be direct or indirect, *via* opsonic intermediates derived from plasma or tissue sources. Macrophage receptors such as CD11b/CD18 can bind *via* opsonic complement components, including secretion products of macrophages themselves, or promiscuously to other ligands, so that it becomes difficult to classify as direct or indirect.

We have outlined the heterogeneity of the macrophage populations involved in clearance, and the complexity of their potential apoptotic/necrotic cargo. It has become customary to break the overall process down into distinct stages: “find me” (36, 37), by chemokine-receptor attractants and plexin–semaphorin guidance/repulsion (38, 39); “eat me” (40) and “don’t eat” me (41) signals between apoptotic cells and phagocytes, involving diffusible products, plasma components such as thrombospondin, and intercellular interactions. We focus here on recognition (42) by macrophages of well-defined apoptotic cells. Apart from the well-documented chromatin and cytoplasmic changes, we consider PS (43) and other potential ligands expressed on the surface of senescent and apoptotic cells and the wide range of direct and indirect opsonic receptors expressed on the macrophage surface (Figure 1). This information is based mainly on *in vitro* studies, with some support obtained from mouse and other genetic models *in vivo*. Phagoptosis, the uptake of viable cells (15), resulting in their destruction, depends on exposure of “eat me” and/or loss of “don’t eat me” signals; it mediates turnover of erythrocytes, neutrophils, and other cells, and also contributes to defense against infection.

Several aspects of our present understanding deserve further comment: there is a bewildering range of possible molecular interactions reported in this literature, depending to a large extent on the interests of the researcher rather than proven relative importance under different conditions *in vivo*. It is perhaps not surprising that so critical a housekeeping function should involve considerable redundancy; it is well known that inhibitors or ablation of favorite individual receptor–ligand pairs are at best only partial in their effect *in vitro*, and often undetectable *in vivo*. For example, this was found in our own studies of possible SR-A involvement in the thymus, a site of avid apoptotic thymocyte clearance by macrophages (44). Up to a point, one could argue that some of these candidates could act in a common pathway or be redundant. A more likely interpretation is that there is organ-specific involvement of particular receptor–ligand pairs, as indicated by new evidence, discussed in more detail below. Furthermore, most previous studies have failed to appreciate the modulation of phagocytic activity by physiological and pathological regulation of particular pathways in different tissue environments. We should expect wide variation from the experimental use of species as divergent as *Drosophila*, zebra fish, mouse, and man, with clearance mechanisms arising by convergent as well as divergent evolution. Finally, since different cell types can perform the same function, constitutively or after induction, it is essential to identify the particular cells involved by cell-specific deletion in any experimental model; for example, this was demonstrated by Ravichandran and colleagues in a receptor transgenic overexpression model which showed enhanced clearance was attributable to epithelial cells and not macrophages, as might have been assumed (45). The macrophage ablation studies during development, e.g., in PU-1 null mice (46) show remarkably little impact except for minor deficiencies in interdigital web regression; presumably, alternative mesenchymal cells can compensate for developmental clearance of apoptotic cells. Failure to clear erythrocyte nuclei, however, does create hemodynamic problems after birth and early death postnatally can also be ascribed to infection.

## Mechanisms of Clearance of Apoptotic Cells by Macrophages

We have previously outlined some of the broad features of opsonic, antibody, and complement-mediated phagocytosis (5), the phagocytic synapse model based on Dectin-1 mediated uptake of yeast particles (47), and a kinetic exclusion model of other plasma membrane molecules during uptake (4); its relevance to the mechanism of apoptotic cell uptake has not been determined. Intravital microscopy has revealed striking recruitment of different mononuclear cell populations during development (48) and following sterile and infectious injury and repair (28). Phosphatidylinositol kinases play an important role in signaling and ingestion of apoptotic cells (49). Various phosphoinositide mediators derive from preformed membrane phospholipids and reorganization of lipids within the plasma membrane bilayer with resultant PS exposure on the outer surface, as elucidated by the groups of Nagata and Tanaka (3) and Kay and Grinstein (50). BAI-1, an adhesion G protein-coupled receptor (51, 52), mediates uptake of apoptotic cells *via* PS recognition, followed by Dock and Elmo signaling. Effects of apoptotic cell uptake on membrane traffic, vesicular fusion and fission, the role of small GTPases, phagosome formation and function (53), as well as possible interactions with autophagy require further investigation. Similarly, there is need for more studies on the effects of apoptotic cell uptake on gene and protein expression, for example, by *in situ* hybridization and multiplex histochemistry, as well as of posttranslational and epigenetic modifications, in heterogeneous cell populations and individual macrophages, *in vivo* and *in vitro*.

## Clearance of Apoptotic Cells by Resident Tissue Macrophages *In Vivo*

It has been known for some time that resident macrophage populations in different organs such as liver, gut, lung, bone marrow, spleen, and brain are heterogeneous in their morphology, turnover, and antigen expression profile. Gene expression studies by Lavin and colleagues have documented organ-specific gene and enhancer patterns, as well as canonical signatures for a range of tissue macrophage populations (54). Apoptotic cell clearance is mediated by distinct pathways in different organs and helps to shape the local phenotype, as shown by recent studies by the groups of Hidalgo (31) and Magarian Blander (30). A-Gonzalez and colleagues used a parabiotic model to study homeostatic leukocyte clearance *in vivo*. Distinct receptors, opsonins, and transcription factors were demonstrated in uptake by macrophages in the bone marrow, spleen, intestine, liver, and lung interstitium; alveolar macrophages in the lung were not accessed by blood delivery. Mutant mice deficient in milk fat globule—EGF factor 8 (lactadherin), liver X receptor transcription factor alpha/beta, peroxisome proliferator activator receptor transcription factor gamma, and proto-oncogene c-mer tyrosine kinase (MerTK) differed from wild-type controls and varied in their contribution to uptake in bone marrow, spleen, and liver. Annexin A1 and Timd4 were also utilized to determine tissue-specific dynamics and expression of different phagocytic mediators. Gene expression analysis demonstrated preservation of distinct core signatures, but phagocytosis imprinted a distinct anti-inflammatory profile, upregulation of CD163 and of CD206, the macrophage mannose receptor, and decrease of the pro-inflammatory expression of IL-1 beta. Expression of CD206 made it possible to identify phagocytic macrophages in the steady state in the absence of experimental manipulation. Clearance activity confirmed the diurnal rhythm of neutrophil turnover. Several interesting aspects of this study require further study: the role of variation in apoptotic rate in neutrophils, the impact of non-circulating apoptotic cells, e.g., generated in the thymus by dexamethasone treatment, and the absence of detectable annexin binding in some experiments, consistent with uptake and subsequent killing of viable cells by phagoptosis. Furthermore, the effects of concurrent inflammation and infection on the clearance of apoptotic as well as necrotic cells, add an additional layer of complexity.

The study by Cummings et al. used a different genetic strategy to study the effects of apoptosis in a single organ, the small intestine, on gene expression by macrophages and DC, after uptake of dying epithelial cells (30). Diphtheria receptor expression in mice was targeted to the intestine by a villin construct with an eGFP reporter; low dose diphtheria toxin induced synchronous apoptosis selectively to ileum epithelium without affecting barrier function. Five subpopulations of APC were isolated before and after apoptosis by FACS, and gene expression was analyzed. Two CD64^+^ macrophages and one CD24^+^ DC population were shown to display distinctive signatures after phagocytosis of apoptotic cells; the macrophage signatures included lipid metabolism and aromatic amino acid catabolism, whereas the DC subset program was consistent with activation of regulatory CD4^+^ lymphocytes. A common signature of suppression of inflammation consisted of distinct genes in macrophages and DCs. Only the DCs were induced by apoptotic intraepithelial cell (IEC) uptake to migrate to draining lymph nodes and found able to induce immunological tolerance. Several of the differentially induced genes in phagocytes overlapped with susceptibility genes for inflammatory bowel disease. CD103^+^CD11b^+^ and distinct CD11b^+^ only macrophages, which sampled the majority of apoptotic IEC^+^, upregulated growth arrest specific protein 6, Cd 163, and MerTK genes, whereas CD103^+^ DC expressed the gene for CD105, a member of the mannose receptor family. Other differentially expressed genes included those implicated in apoptotic cell clearance, enhanced anti-inflammatory, and reduced pro-inflammatory pathways.

A recent study by Roberts et al. (55) demonstrated that selected tissue-resident macrophages in the peritoneal and pleural cavities, and lung alveolar space, are locally programmed for silent clearance of apoptotic cells. Specific populations of macrophages clear apoptotic cells in different tissues; they share features that limit recognition of nucleic acids in response to signals from their local tissue microenvironment. The transcription factor Krüppel-like factor-2 (KLF2), and to a lesser extent KLF4, control the expression of many genes within the macrophage clearance program. These characteristics include high expression of receptors for apoptotic cells such as Tim4, but low expression of toll-like receptor (TLR)9 and reduced TLR responsiveness to nucleic acids. The environmental signals in different tissues, which are unknown, can override inflammatory stimulation and are lost when cells are isolated, *in vitro*. Other tissue-resident macrophages vary in their inhibition of inflammatory responses after apoptotic cell uptake, and it is unclear how homeostasis is maintained in the face of infection, or restored during resolution of inflammation.

Similarly, Baratin et al. reported that a population of CX3CR1^+^CD64^+^MerTK^+^CD11c^+^ resident macrophages, but not CD8alpha DC, were the main local efferocytic cells in the T zone of lymph nodes; however, these scavenger macrophages did not prime CD4 T cells efficiently, unlike DC (56). Tingible body macrophages, prominent in germinal centers in lymph nodes, spleen, and other lymphoid structures, clear apoptotic B lymphocytes. Their possible role in B lymphocyte affinity maturation and antibody diversification and interactions with follicular DCs and other mesenchymal stromal cells require further investigation (57).

## Role of Phagocytic Clearance of Dying Cells in Inflammation and Its Resolution, Tissue Growth, and Immune Regulation

The uptake of apoptotic cells by macrophages is not “silent,” but anti-inflammatory. Secretory mediators include cytokines [transforming growth factor beta (tgf beta), IL-10] and arachidonate metabolites, e.g., prostaglandin E2, as well as a range of proresolving mediators. Addition of macrophages that have ingested early apoptotic cells, to lipopolysaccharide (LPS)-induced inflammatory cells *in vitro* or *in vivo*, reduced inflammatory pathways (20), whether through the above mediators or by undefined direct contact mechanisms. However, genome wide gene expression, proteomic and metabolomic studies may yield a more complex picture of pro- and anti-inflammatory signature markers which should be reinvestigated by newly developed population and single cell methods. *In vitro* studies need to take into account the tolerogenic and pro-inflammatory subtypes of macrophages and DC, the nature of the apoptotic cell targets, clearance receptor usage, and possible association with TLR. Previous exposure to priming agents, including apoptotic cells themselves, can convert the response to a secondary challenge (58). The *in vivo* studies described above have provided evidence for “imprinting,” comparable to “trained immunity” (59), of an anti-inflammatory phenotype in several different tissues, but further studies are needed in the presence of infection or trauma, and associated cell death.

Studies in *Drosophila* (48) and a range of vertebrates have implicated macrophages in the replacement of epithelial and other cells during development and in tissue repair after wounding. Apoptosis and proliferation of microglia are coupled in the adult brain (60). Dying cells themselves are able to generate recruiting and mitogenic signals to attract and interact reciprocally with macrophages. These include a range of trophic factors such as insulin-like growth factor-1 (IGF-1) (45) and TNF-related cytokines (61), released after phagocytic clearance. Extracellular reactive oxygen species (ROS) produced by epithelial disk cells drive apoptosis-induced proliferation *via Drosophila* macrophages (61). The Ravichandran group has shown that IGF-1 released by macrophages in response to uptake of apoptotic cells, can inhibit apoptotic cell clearance by neighboring epithelial cells, while enhancing their uptake of smaller vesicles (45). Ueno et al. showed that CX3CR1^+^ microglia were required for survival of selected cortical neurons during development, ascribed to IGF-1 production after uptake of apoptotic neurons (62). Conversely, Brown and Neher have postulated that microglial phagoptosis mechanisms contribute to neuronal loss, synaptic sculpting, and loss of neuronal processes during development and neurodegeneration (63).

A striking example of the link between phagocytic clearance by macrophages and cell growth and differentiation was demonstrated by Kawane et al. (64). DNAse II in macrophages is responsible for destruction of nuclear DNA from erythroid precursors after enucleation, and essential for definitive erythropoiesis in mouse fetal liver. Recent studies by Fearnhead et al. have shown that Ferroptosis, a regulated physiological cell death process which requires free ferrous iron, occurs through Fe(II)-dependent lipid peroxidation when the reduction capacity of a cell is insufficient (16). Most studies have not distinguished between apoptotic, anti-inflammatory and immunomodulatory, versus pro-inflammatory, necrotic targets, or the source of contact-dependent, vesicular, or soluble products such as ROS and lipid metabolites. The nature of the apoptotic stimulus, for example, programmed versus X-ray-induced cell death, the heterogeneity of mononuclear phagocyte populations, and the local tissue environment can all contribute to the outcome. Most striking is the regenerative capacity of species such as axolotls (65) after limb amputation, for example, which depends on early recruitment and engulfment by phagocytic macrophages (66). A recent comprehensive analysis of gene expression during regeneration of the axolotl limb provides a resource to identify critical genes (67).

Dying cells release a complex mixture of lipids, oxidized phosphoryl choline derivatives, which can display opposing activities, interfering with TLR signaling to prevent inflammatory responses, while hyperactivating DCs and macrophages to promote inflammation by IL-1. CD14, the microbial LPS receptor, expressed by monocytes and macrophages, and downregulated by DC, is an early central regulator of both activities (68). Zanoni et al. demonstrated further that isolated lipid constituents of the mixture induce inflammasome-dependent macrophage hyperactivation *via* CD14, without compromising macrophage viability, thus promoting inflammation in sepsis, but not lethality.

**Figure 2:**
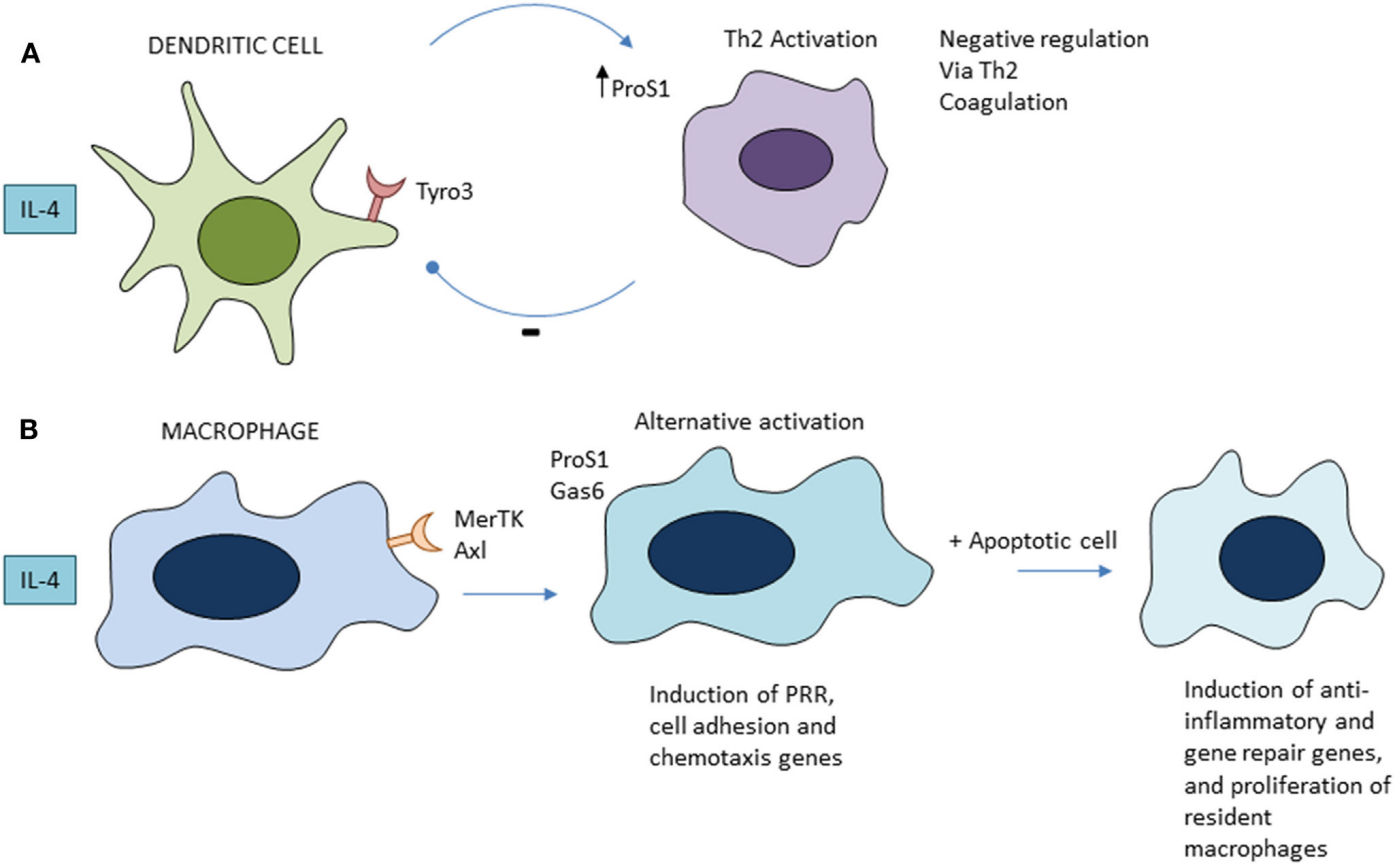
Role of tyrosine receptor kinases Tyro 3, Axl, and MerTK family apoptotic receptors in regulation of Th2 immunity **(A)** and alternative activation of macrophages **(B)**. See text for further details and references.

IL-4 and other Th2 cytokines, possibly of innate sources, induce growth and an alternative activation pathway of macrophages, which has been implicated in wound repair (32). The macrophage mannose receptor (CD206), one of the characteristic signature genes induced by IL-4, is upregulated in mouse models of intestinal apoptosis, in which there is regeneration of epithelium, although it has not been determined whether it is required. A recent study has shown that apoptotic cell phagocytosis selectively amplifies the gene signature induced in macrophages by IL-4/13 (69). The tyrosine receptor kinases Tyro 3, Axl, and MerTK (TAM) receptors and their ligands play distinct immunoregulatory roles in DCs and macrophages (70) (Figure 2). Tyro3 and gene for protein S(eattle) mediate a negative feedback loop between DC and activated Th2 lymphocytes to inhibit type 2 immunity (71); by contrast, Mer-TK and Axl on macrophages require a local apoptotic ligand to promote a full tissue repair program (69). If lysosomal peptides gain access to MHC or cytosol, cross-presentation can induce autoreactive CD8^+^ cytotoxic lymphocytes. Ly6^+^ monocytes are able to efferocytose apoptotic cells and cross-present cell-associated antigens to CD8^+^ T cells; this is enhanced by ligation of TLR7, but not TLR4 (72). Autoreactive Th17 lymphocytes can also be induced by apoptotic cell products in the context of microbial infection (73). Kleinclauss et al. reported that intravenous infusion of apoptotic spleen cells induced a tgf beta-dependent expansion of regulatory T lymphocytes, contributing to tolerance, induced by macrophages and not DCs (74). Saas and colleagues (21) have discussed the mechanism of early apoptotic cell infusion to prevent and limit ongoing autoimmune inflammation.

## Concluding Remarks

Given the importance of the biological process of apoptotic cell clearance, it seems surprising that it might go wrong so infrequently; is there such redundancy and backup to keep it safe, or are we looking in the wrong place? Of course, we know of some autoimmune conditions, e.g., systemic lupus erythematosus (SLE), to which inefficient apoptotic cell clearance contributes; Munoz et al. (75) have discussed the role of nucleic acid and auto-antibody complexes in the pathogenesis of recurrent inflammation, resulting in production of type I interferon, a hallmark of SLE. A major effort is under way to use monoclonal antibodies directed against CD47, a “don’t eat me” signal expressed by selected malignancies, to promote tumor cell ingestion by macrophages (40). A promising, safe protocol has been developed for adoptive transfer of mononuclear phagocytes that have started to engulf apoptotic cells, prophylactically, to facilitate allogeneic hematopoietic transplantation (76). It will be important to characterize the appropriate subpopulation of macrophages for such therapeutic applications. Conversely, we have good reason to believe that uptake of apoptotic HIV-infected lymphocytes by macrophages can provide a route to infection of healthy CD4^+^ T cells (77).

Although the research efforts in this area are mainly based on insights of several decades ago, it would be advisable to use the more advanced methods now available to revisit the fundamental aspects of the phagocytic process. It is exciting to see the recent adoption of intravital methods to study gene expression and growth responses *in vivo*. The holy grail might be to extend the limited regenerative capability of hematopoiesis, intestinal epithelium, and liver more widely to other tissues. In this regard, it would be wise to use a broad comparative approach to discover the factors that shut regeneration down during evolution. Or would that risk enhancing malignant diseases (78)?

## Author Contributions

SG conceived and wrote the manuscript. AP reviewed and edited the manuscript and designed the figures.

## Conflict of Interest Statement

The authors declare that the research was conducted in the absence of any commercial or financial relationships that could be construed as a potential conflict of interest.

## Abbreviations

AIP, apoptosis-induced proliferation; DC, dendritic cell; Gas 6; growth arrest specific protein 6; GPCR, G protein-coupled receptor; IECs, intraepithelial cells; IGF-1, insulin-like growth factor-1; KLF, Kruppel-like factor; LPS, lipopolysaccharide; LXR, liver X receptor transcription factor; MPS, mononuclear phagocyte system; MerTK, proto-oncogene c-mer tyrosine kinase; Megf8, milk fat globule—EGF factor 8 (lactadherin); MNGC, multinucleated giant cell; NET, neutrophil extracellular trap; PGE2, prostaglandin E2; ProS1, gene for protein S(eattle); PRRs, pattern-recognition receptors; PS, phosphatidyl serine; PPAR, peroxisome proliferator activator receptor transcription factor; ROS, reactive oxygen species; SLE, systemic lupus erythematosus; TAM, tyrosine receptor kinases Tyro 3, Axl and MerTK; TLR, toll-like receptor; TNF, tumour necrosis factor; tgf beta, transforming growth factor beta.
